# Bivalirudin Anticoagulant Therapy With or Without Platelet Glycoprotein IIb/IIIa Inhibitors During Transcatheter Coronary Interventional Procedures: A Meta-Analysis

**DOI:** 10.1097/MD.0000000000001067

**Published:** 2015-08-14

**Authors:** Jiabei Li, Shiyong Yu, Dehui Qian, Yun He, Jun Jin

**Affiliations:** From the Institute of Cardiovascular Science, Xinqiao Hospital, Third Military Medical University, Chongqing, China.

## Abstract

The safety and effectiveness of using the direct thrombin inhibitor bivalirudin during transcatheter coronary interventional procedures remains uncertain.

This study aimed to systematically assess anticoagulation with bivalirudin alone or bivalirudin plus glycoprotein (GP) IIb/IIIa inhibitors (bivalirudin-based anticoagulant therapy) in patients undergoing percutaneous coronary intervention (PCI) procedures by a meta-analysis of randomized controlled trials (RCTs).

Systematical searches of the MEDLINE, EMBASE, and Cochrane databases were conducted. RCTs comparing bivalirudin-based anticoagulant therapy with a comparable heparin therapy in patients undergoing PCI were eligible. Risk ratios (RRs) with 95% confidence intervals (CIs) served as summary statistics.

A total of 38,096 patients from 17 RCTs were randomized to the bivalirudin group (n = 18,878) or heparin group (n = 19,218) in the meta-analysis. No significant differences in death, myocardial infarction or reinfarction, ischemia-driven revascularization, or in-stent thrombosis were observed between the 2 groups (all *P* > 0.05). Notably, bivalirudin-based therapy showed a highly significant 34% decrease in the incidence of major bleeding (RR = 0.66; 95% CI 0.54–0.81; *P* < 0.001) and a 28% reduction in the need for blood transfusion (RR = 0.72; 95% CI 0.56–0.91; *P* < 0.01). Meta-regression analyses demonstrated that additional administration of GP IIb/IIIa receptor inhibitors (*P* = 0.01), especially eptifibatide (*P* = 0.001) and tirofiban (*P* = 0.002), was likely to increase the major bleeding risk associated with bivalirudin.

Bivalirudin, in comparison to heparin, is associated with a markedly lower risk of major bleeding, and the additional use of GP IIb/IIIa inhibitors may weaken this benefit.

## Abstract 2

Supplemental Digital Content is available in the text

## INTRODUCTION

In patients undergoing transcatheter procedures for the treatment of coronary diseases, the optimal antithrombotic regimens for maximizing clinical efficacy and minimizing the risk of bleeding complications have been widely investigated over the past decade. The relatively new direct thrombin inhibitor bivalirudin, which offers a low bleeding risk, might be promising as an alternative to unfractionated heparin (UFH), which is routinely used during coronary interventional procedures. Before the widespread use of clopidogrel or prasugrel pretreatment, bivalirudin was associated with lower incidences of periprocedural major bleeding as well as ischemic outcomes compared to UFH.^1^ Subsequently, the widely recommended oral dual antiplatelet therapy (clopidogrel or prasugrel and aspirin) seemed to weaken the benefit of bivalirudin, which was considered to be a significant decrease in bleeding risk without better clinical efficacy.^2^ Recently, the addition of platelet glycoprotein (GP) IIb/IIIa receptor inhibitors to anticoagulant therapy during transcatheter procedures has provided a clinical benefit of reducing ischemic outcomes.^3–5^ However, in conjunction with antiplatelet agents, the efficacy and safety of bivalirudin relative to UFH have not been well established. A previous meta-analysis compared bivalirudin mono- or bivalirudin-based (bivalirudin plus routine or provisional GP IIb/IIIa inhibitors) anticoagulant therapy versus heparin-based anticoagulation (UFH plus routine or provisional GP IIb/IIIa inhibitors) in patients undergoing percutaneous coronary intervention (PCI).^6^ However, the influence of the adjunctive use of GP IIb/IIIa inhibitors and other important clinical factors on ischemic and bleeding endpoints was not defined in the study. Recently, 2 meta-analyses investigated the clinical utility of bivalirudin versus UFH during PCI without planned use of GP IIb/IIIa inhibitors^7^ and only with the use of GP IIb/IIIa inhibitors,^8^ respectively. Neither study comprehensively showed the efficacy and safety profile of bivalirudin in patients undergoing coronary interventional procedures. Additionally, more recently reported results of several new trials and longer-term observations from previous trials can potentially contribute to the development of antithrombotic therapy during the procedures.^9–12^ We therefore performed a meta-analysis of randomized controlled trials (RCTs) to systematically evaluate the efficacy and safety of bivalirudin mono- or bivalirudin-based anticoagulant therapy in patients undergoing PCI. Meanwhile, the effects of additional use of GP IIb/IIIa inhibitors and other clinical factors on ischemic and bleeding outcomes were also investigated in the meta-analysis.

## METHODS

### Literature Review

A computerized literature search was conducted of studies published from January 1990 through January 2015 in the MEDLINE, EMBASE, and Cochrane Central Register of Controlled Trials databases using the following search terms: bivalirudin, hirulog, heparin, low-molecular-weight heparin, unfractionated heparin, UFH, coronary artery/heart disease, myocardial infarction, acute coronary syndrome, unstable angina, angioplasty, percutaneous coronary intervention, PCI, invasive strategy, randomized, and human. In addition, an extensive manual searching was also performed using cross-references from the eligible articles and relevant reviews. The search was restricted to English-language literature.

### Study Eligibility

RCTs were eligible for inclusion if they compared the efficacy or safety of bivalirudin mono- or bivalirudin-based anticoagulant therapy with comparable heparin therapy during PCI and reported clinical outcomes of interest. Bivalirudin/heparin-based regimens were defined as anticoagulation with bivalirudin/heparin (UFH or low-molecular-weight heparin) plus planned or provisional GP IIb/IIIa inhibitors (eg, abciximab, tirofiban, or eptifibatide). Subgroup analyses within the eligible trials were excluded. Moreover, articles published before the year 2000 and those in the form of study designs, editorials, and reviews also were excluded.

### Data Extraction and Quality Assessment

Two investigators (JL and SY) reviewed all the citations in duplicate to identify eligible studies and independently conducted data extraction and quality assessment using a standardized approach. Data regarding ischemic outcomes (eg, death, nonfatal myocardial infarction or reinfarction, ischemia-driven revascularization, or in-stent thrombosis) and bleeding complications (eg, major bleeding or blood transfusion) were extracted from each of the eligible studies. The reviewers resolved differences through consensus, and any disagreements were resolved by the principal investigator of the present study (JJ). All eligible trials were assessed by the following quality criteria recommended by the Cochrane Collaboration: sequence generation of the allocation; concealment of allocation; blinding of participants, personnel, and outcome assessors; use of intention to treat analysis; and description of withdrawals and dropouts. In addition, the Jadad scale,^13^ a numerical score between 0 and 5, was used to qualitatively assess the quality of the included studies.

### Data Synthesis and Analyses

We used risk ratios (RRs) with 95% confidence intervals (CIs) to express the combined results of individual studies. The pooled effects were calculated according to the Mantel–Haenszel random effects model. For studies with no event of interest in a treatment group, 1.0 was added to all cells for continuity correction.^14^ Heterogeneity across studies was quantified using the I^2^ statistic. I^2^ values greater than 25%, 50%, and 75% were considered evidence of low, moderate, and severe statistical heterogeneity, respectively. Sensitivity analyses, in which the pooled estimates were recalculated omitting 1 study at a time, were conducted to assess the impact of individual studies on the summary estimate of effect. Subgroup analyses were performed to assess the impacts of anticoagulant regimens, clinical settings, invasive strategies, and follow-up duration on overall estimates. Meta-regression analyses were also performed to determine the influences of clinical and demographic factors on the overall results. We assessed publication bias using a Begg funnel plot.^15^ Pooling analyses were performed with the RevMan 5.2 software (The Cochrane Collaboration, Copenhagen, Denmark), and meta-regression analyses were conducted with STATA 10.0 software (Stata Corp., College Station, TX). The results were considered statistically significant at *P* < 0.05 (2-sided). The study was performed in compliance with the Quality of Reporting of Meta-analyses (QUOROM) guidelines.^16^

## RESULTS

### Study Selection and Characteristics

**FIGURE 1:**
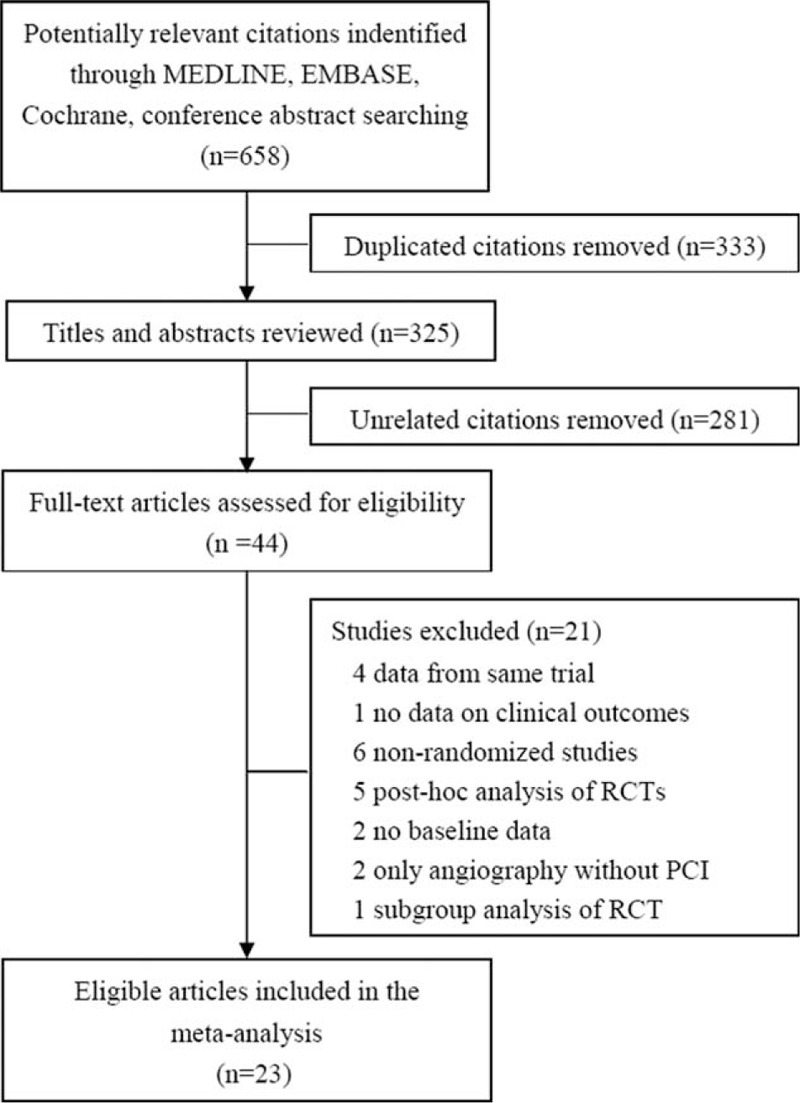
Flowchart of selection of studies for inclusion in meta-analysis. RCTs = randomized controlled trials, PCI = percutaneous coronary intervention.

The process of study selection is illustrated in Figure 1. The electronic searches identified 658 items. After removing the duplicates, we initially screened 325 citations, of which 281 were excluded upon reviewing the titles and abstracts. Forty four potentially eligible studies were scrutinized further by elaborative review of the full text. Finally, 23 articles^10–12,17–36^ involving 17 RCTs were eligible for the final analysis (Figure 1).

A total of 38,096 patients included in the present study was randomized to the bivalirudin-treatment group (n = 18,878; 49.6%) or UFH-treatment group (n = 19,218; 50.4%). The study and demographic characteristics are shown in Tables 1 and 2, respectively. Among the included 17 trials, 3^20,21,26^ compared bivalirudin monotherapy versus UFH monotherapy, 3^18,22,33^ compared bivalirudin versus UFH with planned GP IIb/IIIa inhibitors, 5^10,11,19,30,35^ compared bivalirudin versus UFH with provisional GP IIb/IIIa inhibitors, and 6^12,24,28,29,32,36^ compared bivalirudin monotherapy or bivalirudin plus provisional GP IIb/IIIa inhibitors versus UFH with planned GP IIb/IIIa inhibitors. Five trials^12,18,21,29,33^ focused on patients with non-ST segment elevation acute coronary syndrome, three^10,24,36^ on patients with ST-segment elevation myocardial infarction, and nine^11,19,20,22,26,28,30,32,35^ on patients with unselected coronary heart diseases. Fourteen trials focused on patients undergoing elective PCI, and three^10,24,36^ on those undergoing primary PCI. Six trials^20–22,29,30,35^ reported in-hospital outcomes, thirteen^10,11,17,19,20,23,25,27,28,31,33,35,36^ reported 30-day outcomes, three^20,21,32^ reported 6-month outcomes, five^12,18,26,32,35^ reported 12-month outcomes, and only one^24^ reported 36-month outcomes (Table 1 ). All the included trials reported clinical events of all-cause death, myocardial infarction or reinfarction, or major bleeding, and a composite outcome of death, myocardial infarction or reinfarction, and revascularization. The mean age of patients in the individual trials ranged from 58 to 70 years, and most participants were male (65.1% to 83.4%). The incidence of diabetes ranged from 13% [how effective are antithrombotic therapies in primary percutaneous coronary intervention (HEAT-PPCI)]^10^ to 100% [novel approaches for preventing or limiting events (NAPLES)],^28^ and the prevalence of previous myocardial infarction ranged from about 11% [the harmonizing outcomes with revascularization and Stents in acute myocardial infarction (HORIZONS-AMI)]^23^ to 45% (NAPLES).^28^ Transcatheter procedures were performed in the individual trials mainly through transfemoral access except for HEAT-PPCI trial^10^ (Table 2). In addition, all patients received contemporary evidence-based medical therapy. Postprocedural antiplatelet therapy included aspirin (80–325 mg/day) indefinitely and/or clopidogrel (75 mg/day) for at least 6 to 12 months. The level of evidence for each article was graded with a score of 2 to 5 according to the Jadad quality score (eTable 1, http://links.lww.com/MD/A342).

**TABLE 1:**
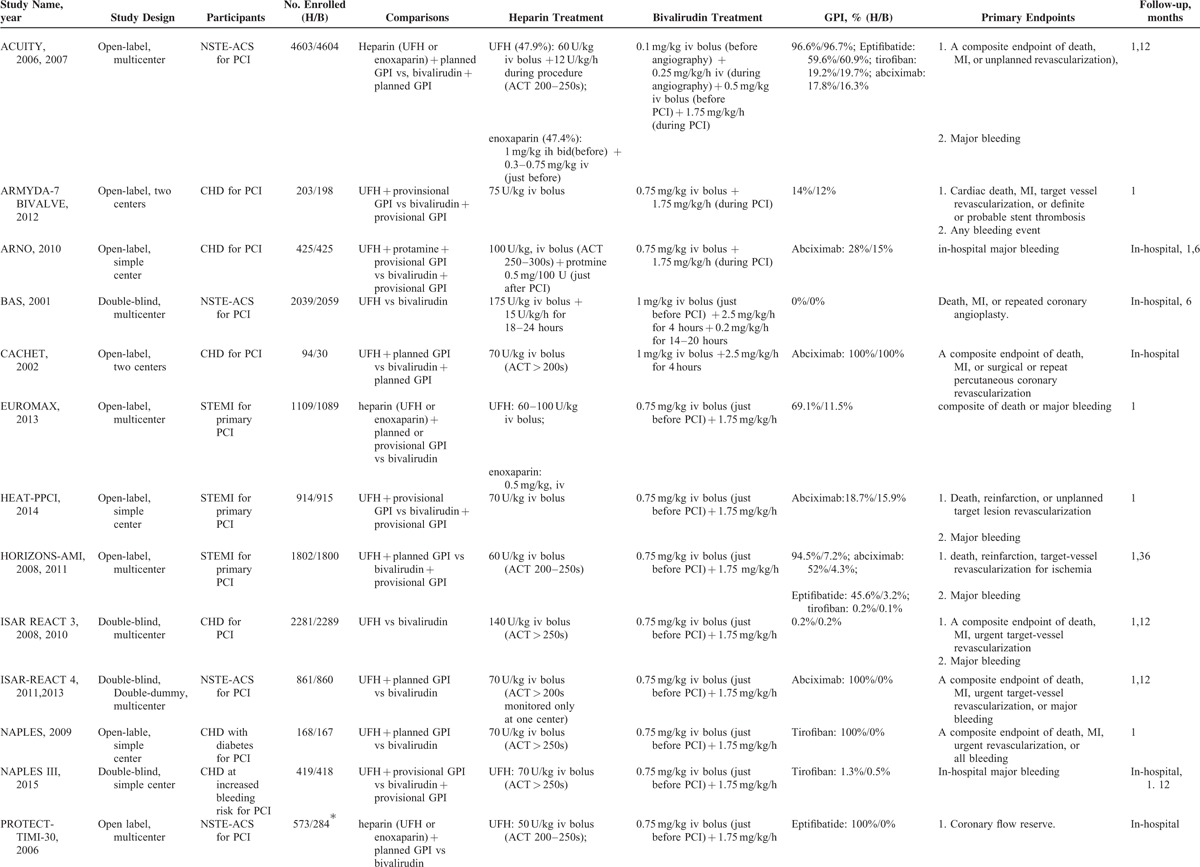
Baseline Characteristics of Studies Included in the Meta-Analysis

**TABLE 1 (Continued):**
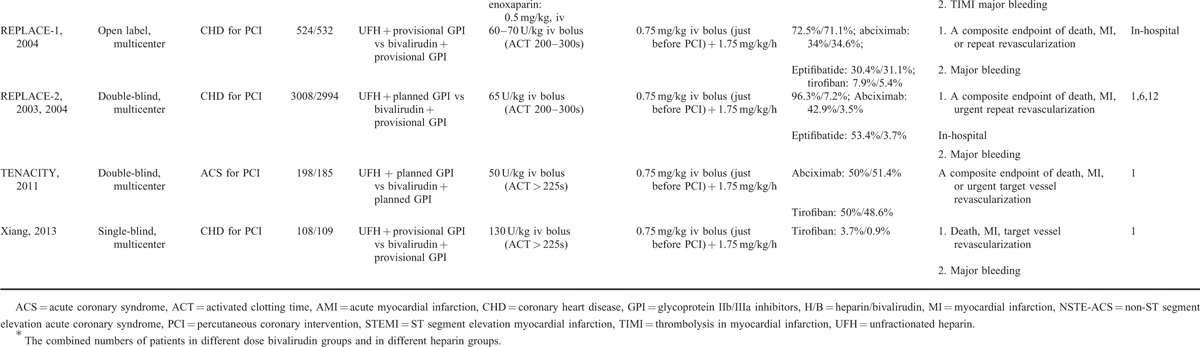
Baseline Characteristics of Studies Included in the Meta-Analysis

**TABLE 2:**
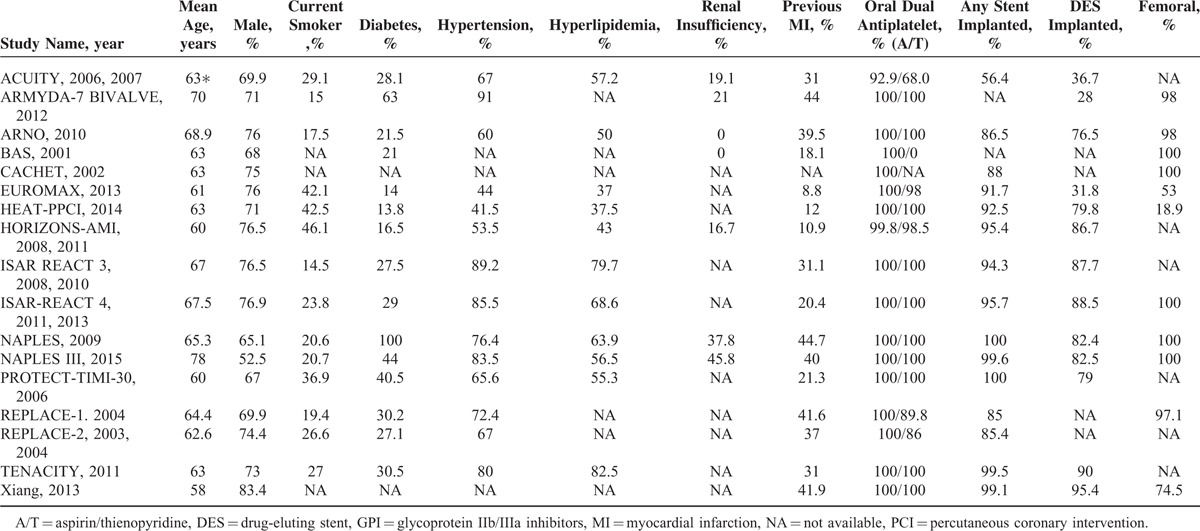
Demographic and Clinical Characteristics of Studies Included in the Meta-Analysis

### Composite Outcomes

The pooled analysis showed that bivalirudin was associated with a similar rate of the composite endpoint as compared with UFH (RR = 1.01; 95% CI 0.94–1.08; *P* = 0.85; I^2^ = 42%). Moreover, the neutral finding was also consistently found in subgroup analyses regardless of anticoagulant regimens, clinical settings, or follow-up duration (Table 3). Additionally, meta-regression analyses did not reveal a substantial influence of clinical or demographic factors on the results (all *P* > 0.05; eTable 2, http://links.lww.com/MD/A342).

**TABLE 3:**
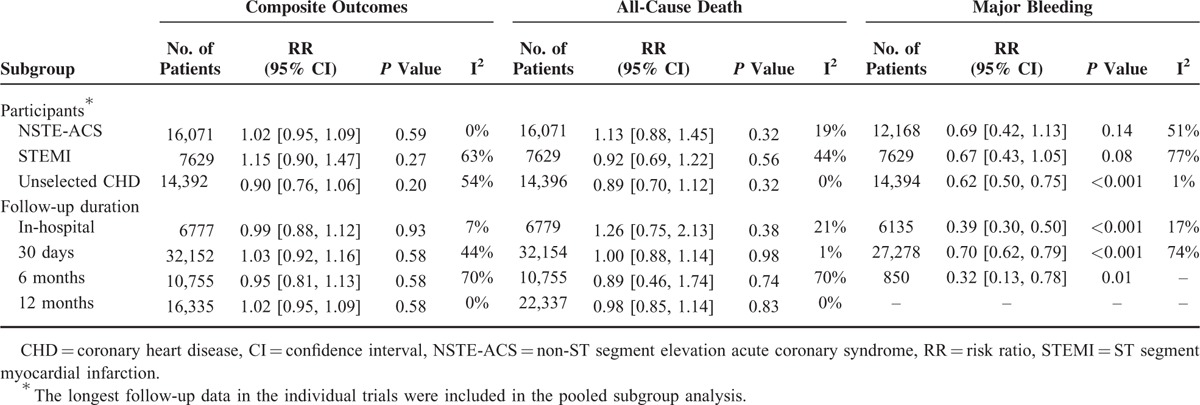
Subgroup Analyses

### All-Cause Death

**FIGURE 2:**
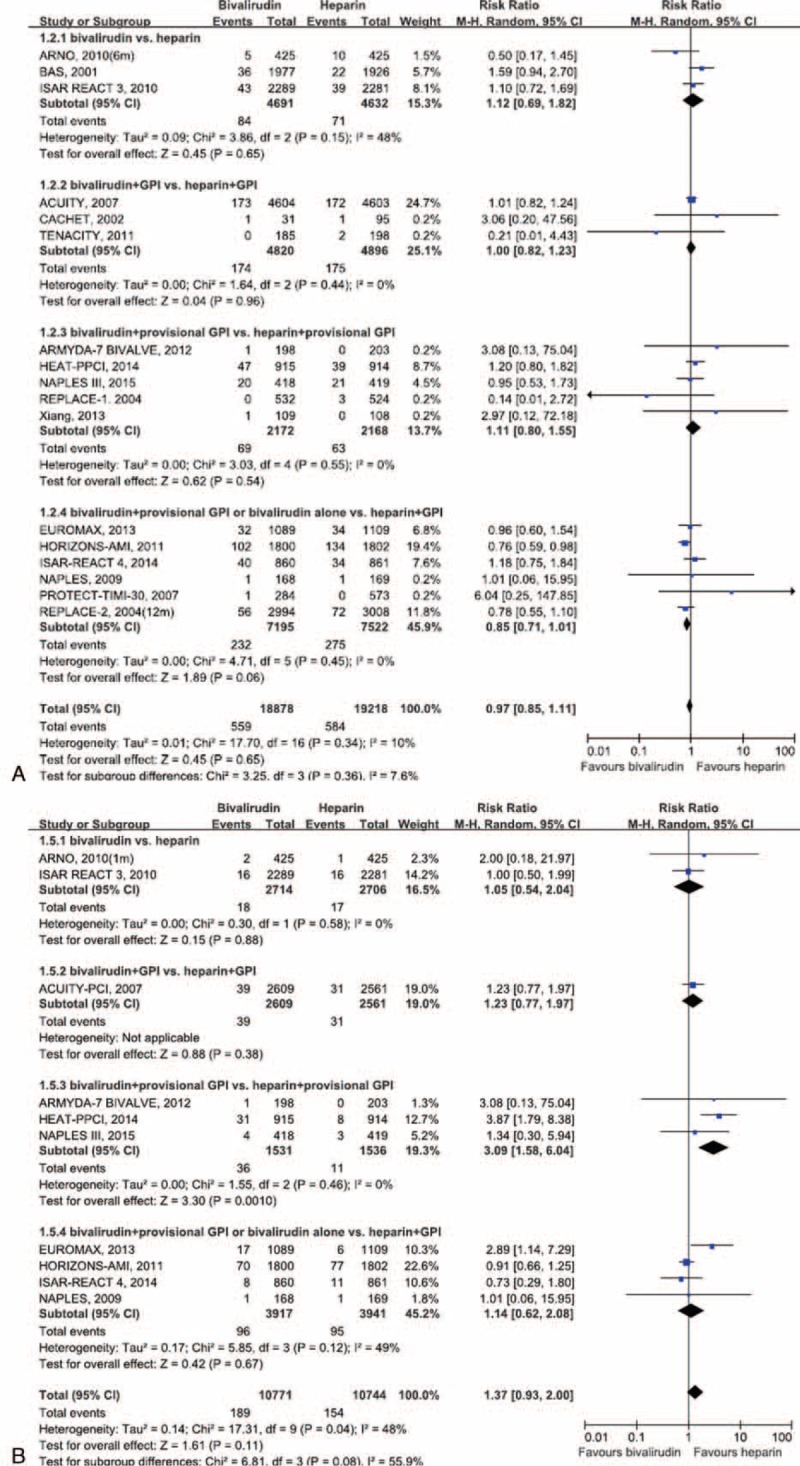
Pooled risk ratio of bivalirudin versus heparin for all-cause mortality (A) and in-stent thrombosis (B). CI = confidence interval.

Overall, 559 of 18,878 patients died from all causes in the bivalirudin-treatment group compared with 584 of 19,218 patients in the UFH-treatment group, with no significant difference between the groups (RR = 0.97; 95% CI 0.85–1.11; *P* = 0.65; I^2^ = 10%; Figure 2A). Moreover, subgroup analyses stratified by anticoagulant regimens did not reveal statistically significant differences in all-cause mortality between the 2 groups (all *P* > 0.05). However, when the intracoronary stenting and antithrombotic regimen–rapid early action for coronary treatment (ISAR-REACT) 4 study^12^ or the evaluate the relative protection against post-PCI microvascular dysfunction and post-PCI ischemia among anti-platelet and anti-thrombotic agents-thrombolysis in myocardial infarction-30 (PROTECT-TIMI-30) study^29^ were removed from the subgroup of bivalirudin alone or bivalirudin plus provisional GP IIb/IIIa inhibitors versus UFH plus planned GP IIb/IIIa inhibitors, we found that the intrasubgroup difference became statistically significant (*P* = 0.02 and 0.045, respectively). Nevertheless, this process did not markedly influence the overall estimate. Moreover, in subgroup analyses and meta-regression analyses, the predefined clinical factors did not have statistically significant influences on the pooled result (Table 3 and eTable 2, http://links.lww.com/MD/A342).

### Myocardial Infarction or Reinfarction, Ischemia-Driven Revascularization, and In-Stent Thrombosis

Meta-analytic pooling for myocardial infarction or reinfarction, ischemia-driven revascularization, and in-stent thrombosis showed that bivalirudin did not provide a greater advantage relative to UFH (myocardial infarction or reinfarction: RR = 1.02; 95% CI 0.91–1.16; *P* = 0.70; I^2^ = 39%; ischemia-driven revascularization: RR = 1.03; 95% CI 0.92–1.15; *P* = 0.58; I^2^ = 40%; and in-stent thrombosis: RR = 1.37; 95% CI 0.93–2.00; *P* = 0.11; I^2^ = 48%; Figure 2B). Subgroup analyses stratified by anticoagulant regimens demonstrated that bivalirudin plus provisional GP IIb/IIIa inhibitors seemed likely to increase the risk of in-stent thrombosis compared with UFH plus provisional GP IIb/IIIa inhibitors (RR = 3.09; *P* < 0.001; Figure 2B). Notably, the HEAT-PPCI study^10^ was likely to greatly contribute to the negative result, because the statistical difference disappeared after the removal of this study from the subgroup.

### Major Bleeding and Blood Transfusion

**FIGURE 3:**
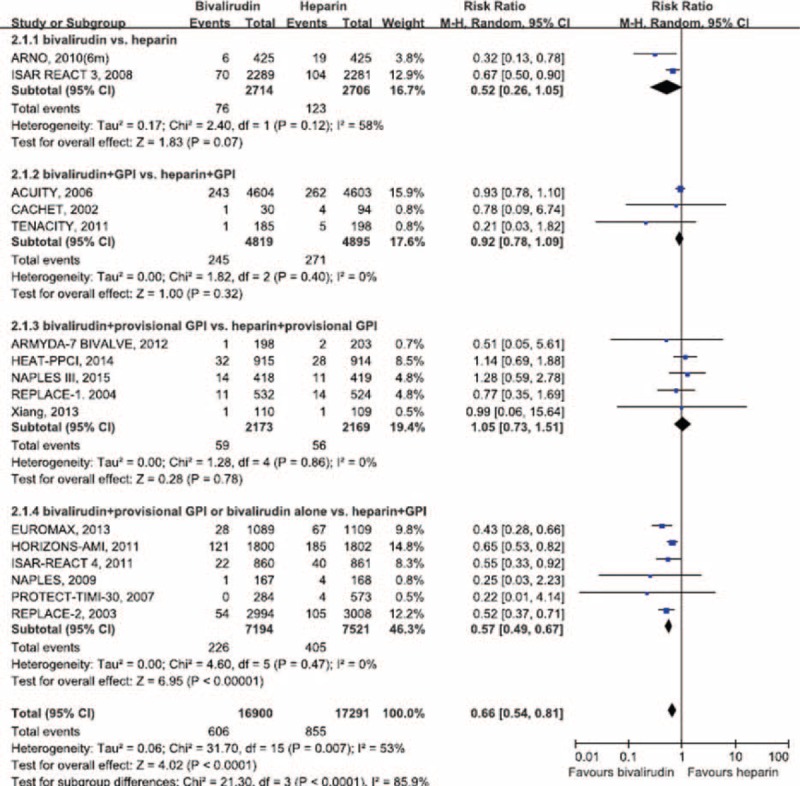
Pooled risk ratio of bivalirudin versus heparin for major bleeding. CI = confidence interval.

**FIGURE 4:**
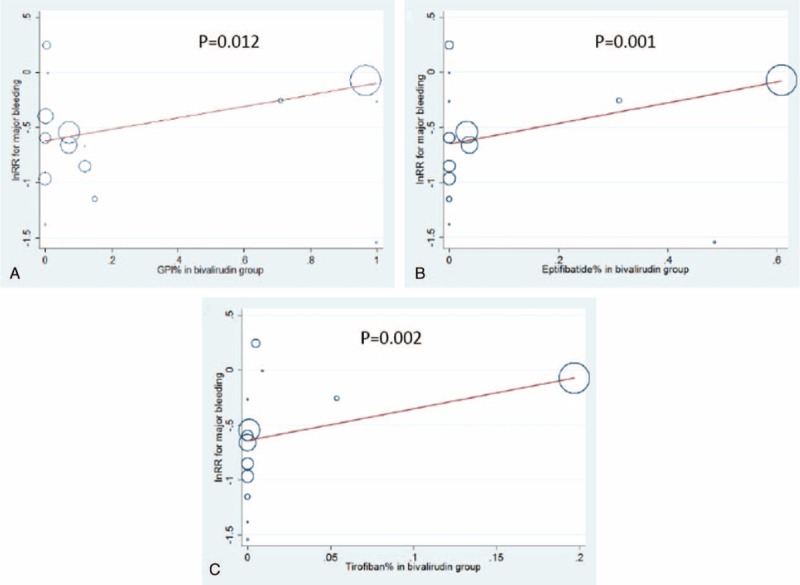
Meta-regression analyses of major bleeding based on the use frequency of all GP IIb/IIIa inhibitors (A), eptifibatide (B), or tirofiban (C) in the bivalirudin group, and that of provisional GP IIb/IIIa inhibitors in the 2 groups (D).

Bivalirudin showed a highly significant 34% decrease in the incidence of major bleeding (RR = 0.66; 95% CI 0.54–0.81; *P* < 0.001; I^2^ = 53%; Figure 3) and a 28% reduction in the need for blood transfusion (RR = 0.72; 95% CI 0.56–0.91; *P* < 0.01; I^2^ = 39%) compared with UFH. Moreover, the benefit of bivalirudin in lowering the risk of major bleeding and subsequent need for blood transfusion was statistically significant in the subgroup of bivalirudin alone or bivalirudin plus provisional GP IIb/IIIa inhibitors versus UFH plus planned GP IIb/IIIa inhibitors (*P* < 0.01). Furthermore, the beneficial effect of bivalirudin was consistently shown in the subgroup analyses stratified by follow-up duration (*P* < 0.05; Table 3). Notably, the bleeding risk with bivalirudin appeared to increase gradually and significantly with the increase in the use of GP IIb/IIIa inhibitors (lnRR = 0.52; *P* = 0.012, Figure 4A), especially eptifibatide (*P* = 0.001, Figure 4B) and tirofiban (*P* = 0.002, Figure 4C, eTable 2, http://links.lww.com/MD/A342).

There was no evidence for publication bias among the included studies. Funnel plots were generated for the composite endpoint, all-cause death, and major bleeding, and essential symmetries were found. Begg tests based on these data did not show any statistical significances (all *P* > 0.10; eFigure, http://links.lww.com/MD/A342).

## DISCUSSION

This meta-analysis mainly showed that bivalirudin mono- and bivalirudin-based anticoagulant therapies were associated with a lower bleeding risk compared with UFH therapy. The use of GP IIb/IIIa inhibitors may weaken the benefit of bivalirudin in reducing the bleeding risk. In addition, bivalirudin, in comparison to UFH, did not significantly increase the incidence of the individual and composite ischemic endpoints of all-cause death, myocardial infarction or reinfarction, and ischemia-driven coronary revascularization as well as in-stent thrombosis.

The combination of a potent anticoagulant (heparin or bivalirudin) with antiplatelet therapy (aspirin, clopidogrel, or GP IIb/IIIa inhibitors) is routinely used during transcatheter coronary interventional procedures. Recently, the use of bivalirudin as a specific and reversible direct thrombin inhibitor is gradually increasing in order to overcome the limitations encountered with heparin during coronary interventional procedures.^37^ Bivalirudin carries no risk of heparin-induced thrombocytopenia, does not require a binding cofactor such as antithrombin III, and does not activate platelets.^38^ Pharmacologically, these characteristics make bivalirudin an ideal alternative to heparin, especially in patients with antithrombin III deficiency or relatively low platelet levels. Indeed, the present meta-analysis indicated the favorable effect of bivalirudin on lowering the bleeding risk and transfusion rate compared with UFH, and the benefit remained consistent in different observation periods. In the era of antiplatelet monotherapy or dual antiplatelet therapy, a growing body of evidence has identified the beneficial effect of bivalirudin on bleeding risk in patients undergoing transcatheter coronary procedures.^21,25^ However, under the conditions of the present wide use of GP IIb/IIIa inhibitors, it remains uncertain whether bivalirudin is able to exert an identical beneficial effect. The present study mainly investigated the impact of additional GP IIb/IIIa inhibitors on major bleeding associated with bivalirudin or heparin anticoagulant therapy. Unexpectedly and interestingly, we found that the use of GP IIb/IIIa inhibitors, especially eptifibatide or tirofiban, substantially reduced the superiority of bivalirudin over UFH. Specifically, with the increase in the frequency of GP IIb/IIIa inhibitor administration during coronary interventional procedures, the benefit of bivalirudin relative to heparin in lowering the bleeding risk was gradually weakened. That is, under conditions of triple antiplatelet therapy (aspirin, clopidogrel, and GP IIb/IIIa inhibitors), bivalirudin treatment might result in a bleeding risk almost identical to that of UFH therapy, and this result was also identified by our subgroup analyses based on anticoagulant regimens.

Presently, achieving a balance between ischemic outcomes and bleeding events is essential in the field of antithrombotic therapy. Emerging evidence indicates the independent relationship between major bleeding with or without blood transfusion and subsequent death.^39^ Major bleeding may be a powerful predictor of death or poor prognosis in patients undergoing PCI.^40^ The HORIZONS-AMI study,^23^ a prospective randomized trial involving patients with ST-segment elevation myocardial infarction undergoing primary PCI, demonstrated that bivalirudin plus provisional GP IIb/IIIa inhibitors improved the event-free survival at 30 days, mainly due to a significant reduction in major bleeding as compared with that experienced with UFH plus planned GP IIb/IIIa inhibitors. However, the present study did not identify a relationship between bleeding events and ischemic outcomes of all-cause death, myocardial infarction or reinfarction, ischemia-driven revascularization, or in-stent thrombosis. Nevertheless, relative to heparin, bivalirudin did not significantly increase the incidence of composite and individual ischemic outcomes. Moreover, the neutral effect on ischemic outcomes remained highly consistent in our subgroup analyses and meta-regression analyses. Additionally, the present study did not show a pronounced additional influence of GP IIb/IIIa inhibitors on clinical prognosis.

Several limitations of the meta-analysis should be considered. The majority of the included trials did not provide data regarding the precise dose of bivalirudin used. As a result, we did not consider the impact of the bivalirudin dose on its efficacy and safety endpoints, and this meta-analysis still could not confirm whether bivalirudin therapy had a dose-specific effect on ischemic and bleeding outcomes. Moreover, all of the included trials involved the use of clopidogrel, rather than prasugrel or ticagrelor, which are more effective antiplatelet agents for reducing the cardiovascular death/stroke/infarction rate, according to the recommendation for oral dual antiplatelet therapy.^41,42^ Therefore, it remains uncertain whether the use of prasugrel or ticagrelor could change the findings regarding the effect of bivalirudin versus UFH in patients undergoing PCI. In addition, as in other nonpatient-level meta-analyses, the present study utilized summarized published events for each trial as opposed to individual patient data. Nevertheless, the findings in the meta-analysis were generated based on a large-scale population from RCTs, and appropriate meta-analytic techniques with random-effect models were used to pool the effect variables. Moreover, our overall analyses were not influenced by publication bias, and sensitivity analysis further confirmed the credibility of the overall estimates.

In summary, bivalirudin was found to be superior to UFH for reducing the risk of major bleeding and need for blood transfusion, with no increase in the incidence of ischemic outcomes, in patients undergoing PCI. Notably, the adjunctive use of GP IIb/IIIa inhibitors during PCI may weaken the favorable effect of bivalirudin on lowering bleeding risk.

## ACKNOWLEDGMENTS

This work was supported by a grant from the National Natural Science Foundation of China (NSFC) (No. 81470300).
